# Buried Gold

**Published:** 1889-10

**Authors:** 


### Buried Gold.

French statisticians are making a curious calculation of the amount of gold which is annually buried in the United States. M. Victor Meunier asserts after careful inquiries that the American dentists insert in American teeth the enormous amount of 800 kilogrammes (about 1800 lbs.) of the precious metal, which represents nearly 450,000 American dollars. This gold is never recovered, of course, but is buried with the persons in whose mouths it is placed. Making allowance for the rapid increase of the population of the United States and for the continued deterioration of American teeth, it appears that in less than a hundred years American cemeteries will contain a larger amount of gold than now exists in France.

